# GW9508‐Induced Activation of GPR40 in Thymic Epithelial Cells: A Therapeutic Strategy to Delay Thymic Aging

**DOI:** 10.1111/acel.70630

**Published:** 2026-07-13

**Authors:** Qingqing Li, Ping Zhu, Lihong Cui, Fenghua Luo, Yongqin Zhou, Yuanyuan Hu, Chunyan Luo, Shanshan Han, Xie Ding, Maoxi Yao, Honggang Yuan, Yinhong Song

**Affiliations:** ^1^ Hubei Key Laboratory of Tumor Microenvironment and Immunotherapy China Three Gorges University Yichang China; ^2^ Institute of Infection and Inflammation China Three Gorges University Yichang China; ^3^ College of Basic Medical Science China Three Gorges University Yichang China; ^4^ Department of Nephrology The First College of Clinical Medical Science, China Three Gorges University, Yichang Center People's Hospital Yichang China; ^5^ Department of Urology The First College of Clinical Medical Science, China Three Gorges University, Yichang Center People's Hospital Yichang China

**Keywords:** GPR40, GW9508, senescence, T cells, thymic epithelial cells

## Abstract

Age‐associated thymic involution leads to a reduction of T‐cell production, which constitutes a primary factor in immunosenescence, thereby increasing vulnerability to cancer, infections, and autoimmune disorders. Thymic epithelial cells (TECs), essential for T‐cell development, exhibit progressive senescence with aging. The development of strategies to mitigate TECs senescence and delay thymic degeneration has emerged as a significant research focus. Here, aged C57BL/6J mice and immortalized thymic epithelial cells (iTECs) were hired. The marked reduction of GPR40 expression was observed in TECs from aged mice and in senescent iTECs induced by doxorubicin in vitro. Administration of the GPR40 agonist GW9508, antagonist GW1100, or their combination to aged mice or senescent iTECs demonstrated that GPR40 activation effectively restored thymic function in aged mice. Mechanistically, GW9508 targeted GPR40 to elevate intracellular calcium ion levels, thereby activating the AMPK signaling pathway and inhibiting the hyperactivation of the ERK1/2‐MAPK pathway in senescent cells, ultimately enhancing the activity of aged iTECs. Collectively, these findings suggest that the exogenous activation of GPR40 by GW9508 may represent a viable strategy to alleviate thymic senescence and enhance immune function in aged individuals.

## Graphical abstract

GW9508 activates GPR40 to trigger Ca^2+^ influx and AMPKα signaling, repressing ERK1/2‐MAPK cascade. This pathway alleviates thymic epithelial cell senescence and restores T cell production to counteract thymus aging.

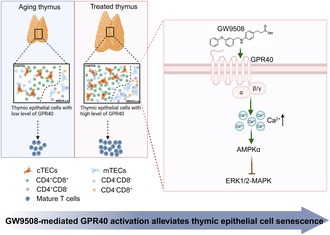

## 1 Introduction

The thymus plays a crucial role in T‐cell development and the establishment of cellular immunity. Thymic epithelial cells (TECs), which constitute the predominant stromal cell population in the thymus, are vital for maintaining thymic structure and function. With aging, the thymus undergoes gradual involution, characterized by a reduction in thymic volume, a decline in TEC numbers, and an accumulation of fibroblasts, adipocytes, and senescent cells within the thymic microenvironment. These changes result in decreased production of naïve T cells and reduced diversity of peripheral T‐cell receptors (TCRs), ultimately compromising immune function in the elderly. Consequently, there is an increased incidence of infectious diseases, tumors, and autoimmune disorders, a phenomenon referred to as immunosenescence (Chinn et al. 2012; Liang et al. 2022). Emerging evidence suggests that TECs within the thymic stroma play a critical role in regulating age‐related thymic involution (Zhu et al. 2007). Therefore, the restoration of both the quantity and functional capacity of TECs may facilitate the recovery of thymic function, potentially yielding significant clinical benefits by enhancing immune competence and preventing or treating age‐associated disorders, malignancies, and infections (Goronzy and Weyand 2019).

GW9508 is a selective agonist of GPR40, a receptor extensively studied in the context of metabolic diseases (Königs et al. 2022; Li, Yu, et al. 2024), and more recently, in relation to age‐associated disorders (Gong et al. 2021; Xiao et al. 2021). GPR40, also known as free fatty acid receptor 1 (FFAR1), is predominantly expressed in pancreatic β‐cells and insulin‐secreting cell lines, as well as in enteroendocrine cells, gustatory cells, immune cells, splenocytes, and the brain (Jin et al. 2024; Puscasu et al. 2025). Within immune system, GPR40 is implicated in regulating the functions of various immune cell, including keratinocytes, macrophages, and neutrophils (Fujita et al. 2011; Souza et al. 2020; Yan et al. 2013). However, the role of GPR40 in senescent TECs has not been documented. Activation of GPR40 is primarily linked to the regulation of intracellular signaling through the G‐protein subunit Gq, which is accompanied by an increase in intracellular calcium levels (Burant 2013; Defossa and Wagner 2014). Here, GW9508, a selective agonist of GPR40, was used to treat aged C57BL/6J mice and aged iTECs model. The results indicated that targeted activation of GPR40 can activate the AMPK signaling pathway while inhibiting the ERK1/2‐MAPK pathway, thereby enhancing the viability and restoring the function of aged iTECs. In vivo experiments in 17‐month‐old mice confirmed the effects of GW9508, consistent with cellular assays results, demonstrating that GW9508 effectively restored thymic function and facilitated structural recovery. Although thymus dysfunction begins relatively early in life, a recent study showed that it retains substantial protective capacity in adults, strongly supporting the notion that enhancing thymus function holds significant potential for improving T‐cell function in older adults (Kooshesh et al. 2023; Wedemeyer et al. 2025). Overall, this study identifies the anti‐senescent properties of GW9508 and elucidates its potential signaling mechanisms, providing novel insights into the pharmacological action of this compound and suggesting a promising therapeutic strategy for mitigating thymic aging.

## 2 Materials and Methods

### 2.1 Mice and Ethical Considerations

Female C57BL/6J mice were procured from the Experimental Animal Center of China Three Gorges University and maintained under specific pathogen‐free (SPF) conditions until they reached 17 months of age. Based on prior research (Gong et al. 2021; Wang et al. 2024), the mice were randomly allocated into five groups: a vehicle group (Dimethyl Sulfoxide, DMSO), a 12.5 mg/kg GW9508 group, a 25 mg/kg GW9508 group, a 50 mg/kg GW9508 group, and a 50 mg/kg GW9508 plus 2.5 mg/kg GW1100 group. The compounds were administered via intraperitoneal injection every other day, totaling 15 doses. Two days after the final administration, upon reaching 18 months of age, the mice were euthanized, and the thymus, spleen, and peripheral blood were collected for subsequent analyses. GW9508 was sourced from TargetMol (No. T1781, Shanghai, China), and GW1100 was obtained from MedChemExpress (MCE, No. HY‐50691, New Jersey, USA). Each control or experimental group comprised a minimum of five animals. All animal procedures were conducted in full compliance with the protocols approved by the Animal Welfare and Research Ethics Committee of China Three Gorges University. Additionally, the study underwent formal ethical review and received approval.

### 2.2 Organ Weighing and Index Calculation

The thymus and body weights of the mice were measured using an analytical balance. The thymus index (Ti) was calculated according to the following formula: Ti = Thymus weight (mg)/body weight (g). The spleen index (Si) was calculated as follows: Si = Spleen weight (mg)/body weight (g).

### 2.3 Cell Culture

The iTECs (Liu et al. 2025; Shen et al. 2020) strains were generously provided by Dr. Jian‐Li Gao's laboratory at Zhejiang Chinese Medical University, Hangzhou, Zhejiang, China. Cells were cultured in Dulbecco's Modified Eagle's Medium (DMEM, PYG0070, Boster, China) supplemented with 10% fetal bovine serum (1101‐18611, Sijiqing, China) at 37°C in a 5% CO_2_ atmosphere. Cells were passaged and plated for drug treatment when they reached a healthy and stable condition. The specific induction method of the senescent cell model is described as follows: Thymic epithelial cells were cultured until fully adherent. Doxorubicin (DOX, 25316‐40‐9, MedChemExpress, USA) was diluted in DMEM complete medium to a final working concentration of 150 ng/mL and added to the cells for 36 h. Successful senescence induction was verified by light microscopy where characteristic senescence‐associated morphological changes (increased cell size, flattened cell shape, and a reduced proportion of cells in the proliferative phase) were observed.

### 2.4 CCK8 Assay

Cells were seeded into 96‐well plates at a density of 6 × 10^3^ cells per well. After cell attachment, 150 ng/mL DOX was added to induce cellular senescence for 36 h. Subsequently, the cells were treated with GW9508 at final concentrations of 0.3 μM, 0.6 μM, 1.2 μM, 2.4 μM, and 4.8 μM, while the control group received an equivalent volume of solvent DMSO. After incubation for 24 h, 48 h, and 72 h, cell viability was assessed using the Cell Counting Kit‐8 (CCK‐8, BS350A, Biosharp, China) according to the manufacturer's instructions. Briefly, diluted CCK‐8 reagent was added to each well, followed by incubation for 1–4 h at 37°C. The absorbance was then measured at 490 nm using a microplate reader. Cell viability was calculated based on the corresponding optical density (OD) values.

### 2.5 Western Blot Assay

Total cellular proteins were extracted using RAPI lysis buffer (BC3711, Solarbio, China). Protein concentrations were determined with a BCA Protein Assay Kit (P1511‐3, Solarbio, China). Equal amounts of protein samples were mixed with loading buffer (G20131, Servicebio, China) and subjected to SDS‐PAGE, followed by electrotransfer onto polyvinylidene difluoride (PVDF) membranes. After blocking with 5% non‐fat milk for 1 h at room temperature, the membranes were incubated with the appropriate primary antibodies overnight at 4°C. On the following day, membranes were washed and incubated with secondary antibodies for 2 h at room temperature. After a final washing step, the protein bands were visualized using a chemiluminescence detection system. The following primary antibodies were used: β‐Actin (GB15001, Servicebio, China), GPR40 (GB112137, Servicebio, China), AMPKα1/2 (WL02254, Wanlei, China), p‐AMPKα1/2 (WL05103, Wanlei, China), ERK1/2 (WL01864, Wanlei, China), p‐ERK1/2 (WLP1512, Wanlei, China), CREB1 (12208‐1‐AP, Proteintech, China), Cyclin D1 (2922, CST, USA), and p21 (2947, CST, USA). The data were analyzed via ImageJ software.

### 2.6 Extraction of Total RNA and Real‐Time RT‐qPCR Analysis

Total RNA was extracted from cells using Trizol reagent (BL4334A, Biosharp, China) according to the manufacturer's protocol. cDNA was synthesized from the isolated RNA using a reverse transcription kit (ABS601512, Absin, China). Quantitative real‐time PCR (qRT‐PCR) was performed using a Bio‐Rad CFX Connect Real‐Time PCR System. The expression levels of target genes were normalized to β‐Actin, and the relative mRNA expression was calculated using the 2^−ΔΔCt^ method. The sequences of all gene‐specific primers used in this study are listed in Table S1.

### 2.7 SA‐β‐Gal Assay

Cellular senescence was assessed using a Senescence β‐Galactosidase Staining Kit (C0602, Beyotime, China) according to the manufacturer's instructions. Briefly, the cells were washed twice with phosphate‐buffered saline (PBS) and fixed with senescence‐associated β‐galactosidase (SA‐β‐Gal) fixation solution for 15 min at room temperature. After fixation, the cells were rinsed with PBS and incubated with freshly prepared SA‐β‐Gal staining solution overnight at 37°C (without CO_2_). The following day, the staining solution was removed, and the cells were washed gently with PBS. Senescent cells exhibiting blue staining were observed under an inverted microscope, and images were captured using a digital imaging system. The activity of SA‐β‐galactosidase was determined by calculating the proportion of positively stained cells.

### 2.8 Flow Cytometry Analysis

Collected cell suspensions were stained with one or more combinations of fluorochrome‐conjugated antibodies according to the experimental design. The antibodies used were as follows: CD3e‐PerCP‐Cy5.5 (551163), CD4‐APC/Cy7 (100413), CD8‐BV510 (100752), CD62L‐APC (S53152), CD44‐BV421 (563970), CD45RB‐PE (553101), PD1‐PE/Cy7 (135215), CD16/32 FcR (101302), CD44‐PerCP/Cy5.5 (560570), CD25‐FITC (102006), C‐kit (CD117)‐PE (553355), FVS‐APC/Cy7 (565388), CD45‐PE/Cy7 (552848), I‐A/I‐E‐BV421 (107631), EpCAM‐PerCP/Cy5.5 (118220), CD80‐BV510 (740130), Ly51‐PE (553735), AIRE‐Alexa Fluor 488 (53593480), and Ki67‐APC (Alexa Fluor 647) (151206). All antibodies were purchased from BioLegend (USA), BD Biosciences (USA), or Invitrogen (USA). After staining, the samples were incubated at 4°C in the dark, washed with PBS or washing buffer, and centrifuged to remove the supernatant. The cells were then resuspended in PBS and analyzed using a flow cytometer (NovoCyte Quanteon, Agilent Technologies, USA). Data analysis was conducted using FlowJo software package (TreeStar Inc., Ashland, USA, version 10.8.1).

### 2.9 Immunofluorescence Staining

Thymic tissues were collected from mice, fixed, dehydrated, and embedded in optimal cutting temperature (OCT) compound to prepare frozen sections. After permeabilization and blocking, the sections were incubated with the corresponding primary antibodies overnight at 4°C. On the following day, the samples were washed and then incubated with secondary antibodies for 1 h at room temperature. Subsequently, the sections were mounted with anti‐fade mounting medium containing DAPI (H‐1200, Vector Laboratories, USA), and fluorescence images were captured using a confocal laser‐scanning microscope. The antibodies used were as follows: GPR40 (GB112137‐50, Servicebio, China), K8 (904,804, BD, USA), and Alexa Fluor 488 (A27034, Thermo Fisher, USA). The average fluorescence intensity was quantified using ImageJ software (National Institutes of Health, Bethesda, MD, USA, version 1.54 p).

### 2.10 Intracellular Calcium Measurement

Cells were collected by digestion with trypsin without EDTA, washed with Hank's balanced salt solution (HBSS; G4203‐500, Servicebio, China), and resuspended in the same buffer. The cell suspension was then incubated with Fluo‐3 AM stock solution (14960, Gayman, USA) at 37°C for 30 min in the dark. After incubation, the cells were centrifuged to remove the residual dye and washed twice with 1 mL HBSS to eliminate excess probe. The cell pellet was resuspended in HBSS and incubated for an additional 20–30 min at 37°C to allow complete intracellular conversion of Fluo‐3 AM to Fluo‐3. Finally, the fluorescence intensity of intracellular calcium was detected using a flow cytometer (FACSVerse, BD, USA); data analysis was conducted using FlowJo software package (TreeStar Inc., Ashland, USA, version 10.8.1).

### 2.11 Analysis of Cell Apoptosis Measurement

Cells were harvested with trypsin digestion, and the cell supernatant was collected simultaneously. After washing twice with pre‐chilled PBS, cells were resuspended in 1× Binding Buffer at a concentration of 1 × 10^6^ cells/mL. The cell suspension was transferred to a new 1.5 mL tube, followed by the addition of Annexin V‐PE and 7‐AAD staining solution (559763, BD, USA). The mixture was gently vortexed and incubated at room temperature in the dark for 15 min. Finally, 200 μL of 1× Binding Buffer was added to each tube, and samples were kept protected from light. All samples were analyzed by flow cytometer within 1 h. Data were analyzed using FlowJo software (TreeStar Inc., Ashland, USA, version 10.8.1).

### 2.12 Cell Transfection

The iTECs in the logarithmic growth phase were seeded into 6‐well plates. Following cell adherence, transfection was performed in accordance with the manufacturer's protocol for the Lipo8000 Transfection Reagent (C0533, Beyotime, China). For si‐*GPR40* transfection, uninduced young iTECs were transfected and subsequently harvested after a 72‐h incubation period for further experimentation. In contrast, for pcDNA3.1‐*GPR40* transfection, induced senescent iTECs were transfected and collected after a 24‐h incubation period for subsequent experimental procedures.

## 3 Results

### 3.1 GW9508 Increased the Thymus Index in Aged Mice

**FIGURE 1:**
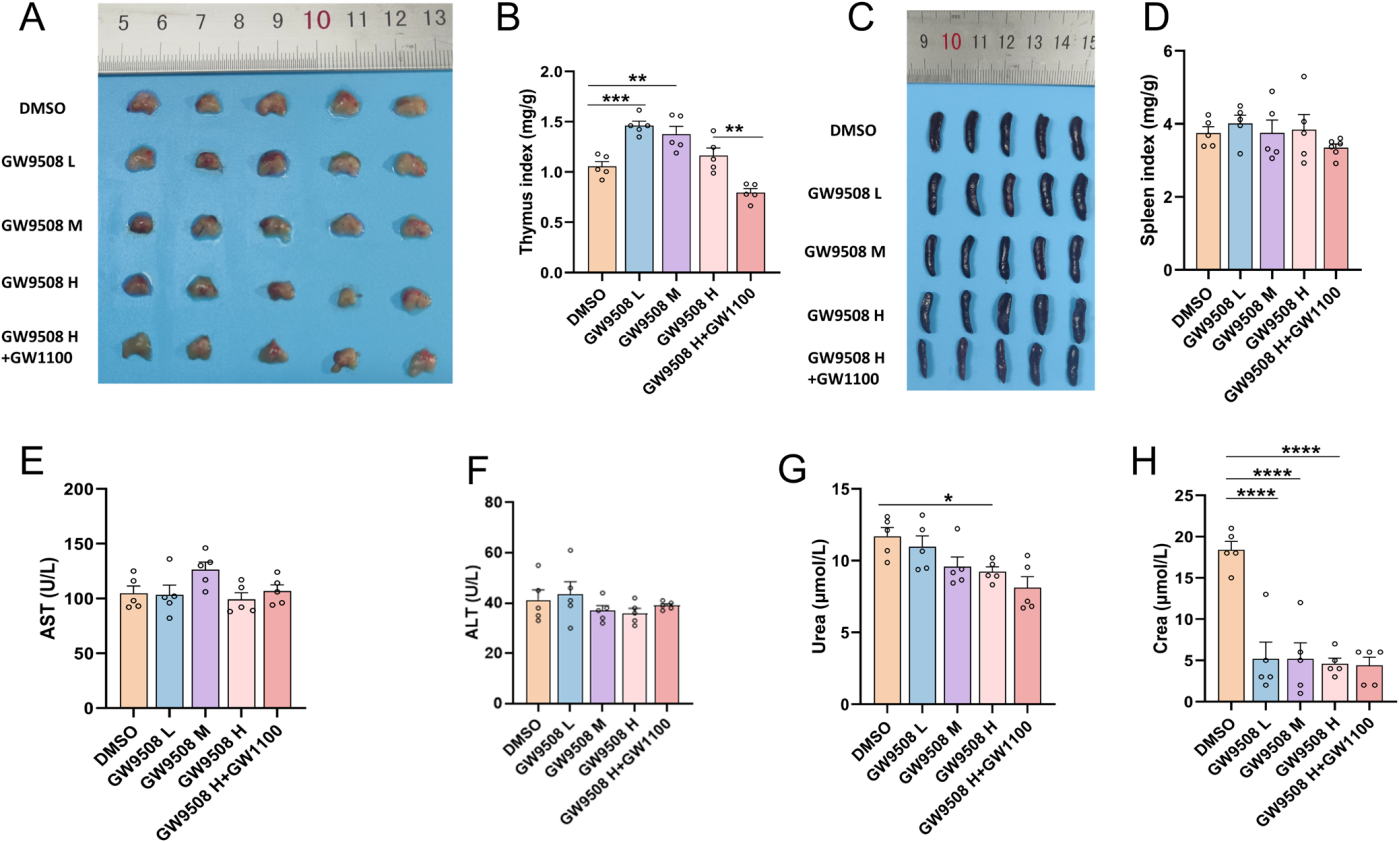
GW9508 increased the thymus index in aged mice. (A, B) Representative gross thymus morphology and quantification of thymic index. (C, D) Representative gross spleen morphology and quantification of the splenic index. (E–H) Serum biochemical analysis of peripheral blood, including AST, ALT, Urea, and Crea levels. Data were presented as mean ± SEM (*n* = 5). One‐way ANOVA with Dunnett's multiple comparison test was performed to compare DMSO, GW9508 L, GW9508 M and GW9508 H groups. Two‐tailed independent samples t‐test was used for comparison between GW9508 H and GW9508 H + GW1100; *p < 0.05, **p < 0.01, ***p < 0.001, ****p < 0.0001.

To investigate the effects of GW9508 on thymic function and its potential to enhance thymocyte development and immune function in aged mice, a study was performed using 17‐month‐old naturally aged female C57BL/6J mice. These mice were randomly assigned to one of five experimental groups: a vehicle control group receiving solvent DMSO, and four treatment groups receiving 12.5 mg/kg GW9508 (L group), 25 mg/kg GW9508 (M group), 50 mg/kg GW9508 (H group), or a combination of 50 mg/kg GW9508 and 2.5 mg/kg GW1100 (H plus GW1100 group). Treatments were administered via intraperitoneal injections every other day for a total of 15 doses. Following treatment, the mice were sacrificed, and thymus, spleen, and peripheral blood samples were collected for analysis. Gross anatomical analysis and thymus and spleen index calculations revealed that GW9508 treatment significantly increased thymus size and the thymus index, particularly in the 12.5 and 25 mg/kg dosage groups, compared to the DMSO control group. In contrast, the co‐administration of GW1100 markedly reduced the thymus index relative to the GW9508 (H group), indicating that GW9508 alleviates age‐related thymic atrophy, while GW1100 exhibits an antagonistic effect (Figure 1A,B). No significant differences in the spleen index were observed among the experimental groups (Figure 1C,D). Biochemical analysis of peripheral blood demonstrated aspartate aminotransferase (AST) and alanine aminotransferase (ALT) levels did not differ significantly between the GW9508‐treated group and the DMSO control group. Interestingly, after GW9508 treatment, the urea level decreased slightly, with a statistically significant reduction observed in the high‐concentration GW9508 group. Meanwhile, the creatinine (Crea) level was markedly reduced. These results indicated that GW9508 exhibits no hepatorenal toxicity in aged mice and may potentially improve renal function in senescent mice (Figure 1E–H).

### 3.2 GW9508 Delayed Thymic Involution and Promoted T Lymphocyte Regeneration in Aged Mice

**FIGURE 2:**
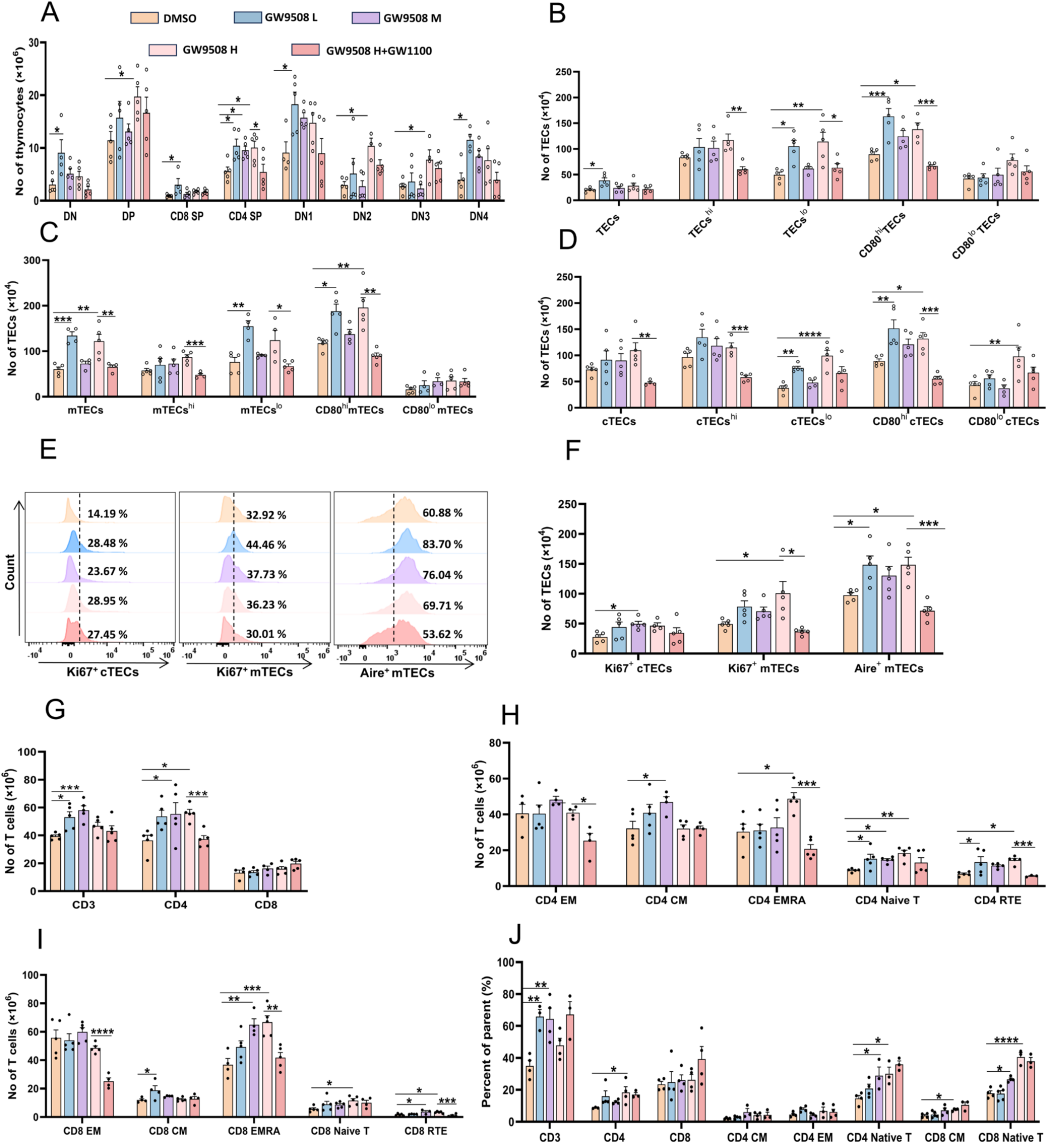
GW9508 delayed thymic involution and promoted T lymphocyte regeneration in aging mice. (A) Quantification of thymocyte numbers for DN (CD4^−^ CD8^−^), DP (CD4^+^ CD8^+^), CD8^+^ SP (CD4^−^ CD8^+^), CD4^+^ SP (CD4^+^ CD8^−^), DN1 (Lin^−^ CD25^−^ CD44^+^), DN2 (Lin^−^ CD25^+^ CD44^+^), DN3 (Lin^−^ CD25^+^ CD44^−^) and DN4 (Lin^−^ CD25^−^ CD44^−^) subsets. (B) Quantification of TECs (CD45^−^ EpCAM^+^), TECs^hi^ (CD45^−^ EpCAM^+^ MHC II^hi^), TECs^lo^ (CD45^−^ EpCAM^+^ MHC II^lo^), CD80^hi^ TECs (CD45^−^ EpCAM^+^ CD80^hi^), and CD80^lo^ TECs (CD45^−^ EpCAM^+^ CD80^lo^) in aged mice by flow cytometry. (C) Quantification of mTECs (CD45^−^ EpCAM^+^ Ly51^−^), mTECs^hi^ (CD45^−^ EpCAM^+^ Ly51^−^ MHC II^hi^), mTECs^lo^ (CD45^−^ EpCAM^+^ Ly51^−^ MHC II^lo^), CD80^hi^ mTECs (CD45^−^ EpCAM^+^ Ly51^−^ CD80^hi^), and CD80^lo^ mTECs (CD45^−^ EpCAM^+^ Ly51^−^ CD80^lo^) in aged mice by flow cytometry. (D) Quantification of cTECs (CD45^−^ EpCAM^+^ Ly51^+^), cTECs^hi^ (CD45^−^ EpCAM^+^ Ly51^+^ MHC II^hi^), cTECs^lo^ (CD45^−^ EpCAM^+^ Ly51^+^ MHC II^lo^), CD80^hi^ cTECs (CD45^−^ EpCAM^+^ Ly51^+^ CD80^hi^), and CD80^lo^ cTECs (CD45^−^ EpCAM^+^ Ly51^+^ CD80^lo^) in aged mice by flow cytometry. (E) Percentage of Ki67^+^ cTECs (CD45^−^ EpCAM^+^ Ly51^+^ KI67^+^), Ki67^+^ mTECs (CD45^−^ EpCAM^+^ Ly51^−^ Ki67^+^), and Aire^+^ mTECs (CD45^−^ EpCAM^+^ Ly51^−^ Aire^+^) in TECs from aged mice analyzed by flow cytometry (Modal processing applied). (F) Quantification of Ki67^+^ cTECs, Ki67^+^ mTECs, and Aire^+^ mTECs in TECs from aged mice by flow cytometry. (G) Quantification of splenic CD3^+^, CD4^+^ (CD3^+^ CD4^+^ CD8^−^), and CD8^+^ (CD3^+^ CD4^−^ CD8^+^) T‐cell subsets in aged mice by flow cytometry. (H) Quantification of splenic CD4^+^ T‐cell subsets (CD4^+^ EM (CD3^+^ CD4^+^ CD8^−^ CD62L^−^ CD44^hi^), CD4^+^ CM (CD3^+^ CD4^+^ CD8^−^ CD62L^+^ CD44^hi^), CD4^+^ EMRA (CD3^+^ CD4^+^ CD8^−^ CD62L^−^ CD44^−^), CD4^+^ naïve (CD3^+^ CD4^+^ CD8^−^ CD62L^+^ CD44^lo^) T cells and CD4^+^ RTE (CD3^+^ CD4^+^ CD8^−^ CD62L^+^ CD44^lo^ CD45RB^lo^) T cells) in aged mice by flow cytometry. (I) Quantification of splenic CD8^+^ T‐cell subsets (CD8^+^ EM (CD3^+^ CD4^−^ CD8^+^ CD62L^−^ CD44^hi^), CD8^+^ CM (CD3^+^ CD4^−^ CD8^+^ CD62L^+^ CD44^hi^), CD8^+^ EMRA (CD3^+^ CD4^−^ CD8^+^ CD62L^−^ CD44 ^−^), CD8^+^ naïve (CD3^+^ CD4^−^ CD8^+^ CD62L^+^ CD44^lo^) T cells and CD8^+^ RTE (CD3^+^ CD4^−^ CD8^+^ CD62L^+^ CD44^lo^ CD45RB^lo^) T cells) in aged mice by flow cytometry. (J) Quantification of CD3^+^, CD4^+^, CD8^+^, CD4^+^ CM, CD4^+^ EM, CD4^+^ naïve T, CD8^+^ CM, and CD8^+^ naïve T‐cell subsets in the peripheral blood of aged mice by flow cytometry. All data were presented as mean ± SEM, *n* = 5. One‐way ANOVA with Dunnett's multiple comparison test was performed to compare DMSO, GW9508 L, GW9508 M and GW9508 H groups. Two‐tailed independent samples *t*‐test was used for comparison between GW9508 H and GW9508 H + GW1100; *p < 0.05, **p < 0.01, ***p < 0.001, ****p < 0.0001.

To evaluate the capacity of TECs in aged mice to support thymocyte development, flow cytometry was employed to analyze the proportions and numbers of thymocytes and their subpopulations. Flow cytometry gating strategy is shown in Figure S1. As shown in Figure 2, GW9508 treatment significantly increased the numbers of double‐negative (DN), double‐positive (DP), CD4^+^ single‐positive (SP), and CD8^+^ SP thymocytes compared to the DMSO control group. Additionally, GW9508 promoted the differentiation of aged thymocytes through all four developmental stages of the DN subset, resulting in increased numbers of DN1, DN2, DN3 and DN4 cells within the thymus. Co‐administration of GW1100 antagonized the effect of GW9508, reversing the observed increases in thymocyte numbers and subpopulation differentiation (Figure 2A). The age‐related decline in TECs and emergence of atypical age‐associated TECs are considered major contributors to the reduced ability of the thymus to generate T cells (Kousa et al. 2024). To evaluate the in vivo effects of GW9508 on TECs, TECs were isolated from the thymi of aged mice using an enzymatic digestion method, and their populations were analyzed via flow cytometry. Flow cytometry gating strategy is shown in Figure S2. As demonstrated in Figure 2, treatment with GW9508 significantly increased the numbers of total TECs, medullary TECs (mTECs), and cortical TECs (cTECs) in aged mice compared to the DMSO control group. Furthermore, GW9508 administration led to increased populations of TECs^hi^, TECs^lo^, CD80^hi^ TECs, CD80^lo^ TECs, mTECs^hi^, mTECs^lo^, CD80^hi^ mTECs, CD80^lo^ mTECs, cTECs^hi^, cTECs^lo^, CD80^hi^ cTECs, and CD80^lo^ cTECs. This findings suggest an enhancement in the thymocyte‐activating and antigen‐presenting capacities of TECs (Figure 2B–D). Additionally, the numbers of Ki67^+^ cTECs, Ki67^+^ mTECs, and Aire^+^ mTECs were significantly elevated, indicating that GW9508 promotes cellular proliferation and antigen presentation, thereby facilitating the negative selection of developing T cells (Figure 2E,F). In contrast, the administration of the GW9508 antagonist GW1100 reversed these effects, demonstrating its antagonistic action.

Mature T lymphocytes, generated in the thymus, migrate to peripheral lymphoid organs including the spleen, which plays a critical role in the peripheral immune system. Within the spleen, T lymphocytes are essential for cellular immune responses (He et al. 2025; Shirafkan et al. 2024). To investigate the effects of GW9508 on peripheral T‐cell immunity, single‐cell suspensions from the spleens of aged mice were prepared, and analyzed via flow cytometry. Flow cytometry gating strategy is shown in Figure S3. As shown in Figur 2, GW9508 administration resulted in a significant increase in the numbers of CD3^+^ T cells and CD4^+^ T cells, although the increase in CD8^+^ T cells was not statistically significant (Figure 2G). Moreover, GW9508 treatment elevated multiple functional subsets within both CD4^+^ and CD8^+^ T‐cell populations, including CD4^+^ effector memory (EM), CD4^+^ central memory (CM), CD4^+^ effector memory RA (EMRA), CD4^+^ naïve T cells, CD4^+^ recent thymic emigrants (RTE), CD8^+^ EM, CD8^+^ CM, CD8^+^ EMRA, CD8^+^ naïve T, and CD8^+^ RTE cells (Figure 2H,I). Recent evidence indicates that an increase in naive CD8^+^ T cells is strongly associated with enhanced longevity and healthspan (Sayed et al. 2021; Youm et al. 2025). These findings suggest that GW9508 mitigates immune senescence and enhanced immune competence in aged mice. Conversely, the administration of GW1100 reversed these beneficial effects, further demonstrating its antagonistic action. Peripheral blood T lymphocytes are essential components of the immune system, with their quantity and functional status serving as key indicators of the overall immune competence. To investigate the effects of GW9508 on peripheral T‐cell populations, flow cytometry was employed to analyze T cells and their subsets in the peripheral blood of aged mice. Flow cytometry gating strategy is shown in Figure S4. As illustrated in Figure 2, administration of GW9508 increased the numbers of CD3^+^ T cells and CD4^+^ T cells compared to the DMSO control group, while the increase in CD^+^ T cells was not statistically significant. Furthermore, GW9508 treatment enhanced multiple functional subsets within both CD4^+^ and CD8^+^ T‐cell populations, including effector memory (EM), central memory (CM), and naïve T cells, with particularly notable increases in CD4^+^ naïve and CD8^+^ naïve T cells (Figure 2J). Previous studies have established a strong link between thymic involution and immunosenescence, with a reduction in CD8^+^ naïve T‐cell frequency being a hallmark of age‐related immune decline (Sandstedt et al. 2023). Consistent with these findings, the results of our study suggest that GW9508 alleviates immune aging in mice, potentially by preserving thymic output and maintaining peripheral naïve T‐cell homeostasis.

### 3.3 GW9508 Enhanced the Viability of Senescent iTECs

**FIGURE 3:**
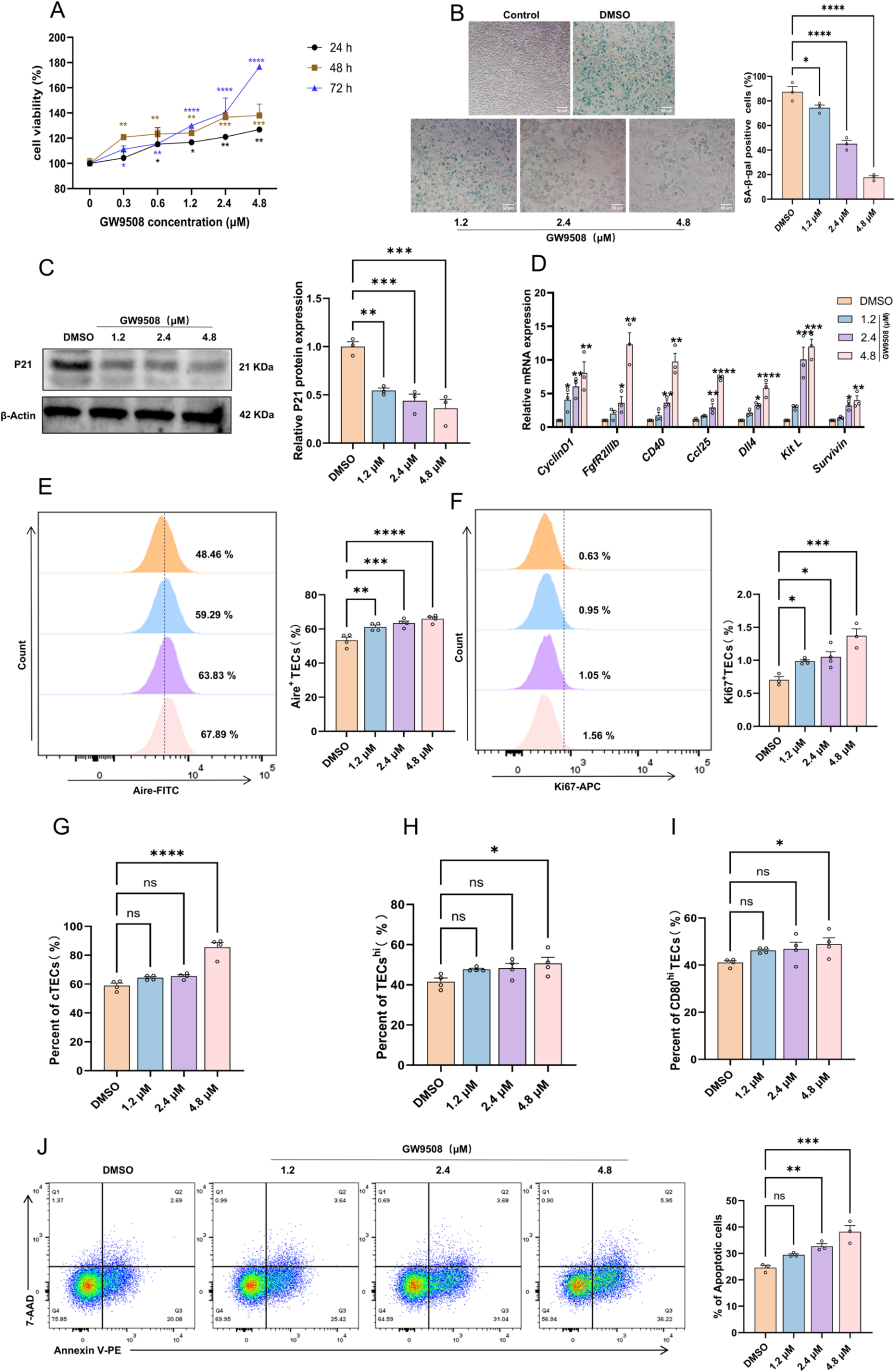
GW9508 enhanced the viability of senescent iTECs. (A) CCK‐8 assay showing cell viability of senescent cells treated with 0.3, 0.6, 1.2, 2.4, and 4.8 μM GW9508 for 24, 48, and 72 h. (B) Representative images of SA‐β‐gal staining and quantification of SA‐β‐gal positive cells following GW9508 treatment for 72 h. Scale bar = 50 μm. (C) Representative Western blot images showing P21 expression in senescent cells after GW9508 treatment for 72 h. (D) RT‐qPCR analysis of functional gene expression in senescent cells after GW9508 treatment for 72 h. (E) Representative flow cytometry plots and quantitative analysis of Aire expression in senescent cells after GW9508 treatment for 72 h. (F) Representative flow cytometry plots and quantitative analysis of Ki67 expression in senescent cells after GW9508 treatment for 72 h. (G‐I) The quantitative analysis of changes in TECs' subpopulations including cTECs (CD45^−^ EpCAM^+^ Ly51^+^), TECs^hi^ (CD45^−^ EpCAM^+^ MHC II^hi^), and CD80^hi^ TECs (CD45^−^ EpCAM^+^ CD80^hi^) after GW9508 treatment for 72 h examined by FCM. (J) FCM was used to detect the apoptosis of senescent cells after GW9508 treatment, and the statistical graph presented the total apoptotic rate of cells in each group. All data are presented as mean ± SEM, *n* = 3. Statistical analysis was performed using one‐way ANOVA with Dunnett's multiple comparison test; ns, *p* ≥ 0.05; *p < 0.05, **p < 0.01, ***p < 0.001, ****p < 0.0001.

To establish a cellular senescence model, iTECs were treated with DOX, leading to a significant reduction in cell viability. Subsequent treatment with GW9508 notably restored the viability of senescent iTECs in a dose‐dependent manner. The most pronounced effect was observed after 72 h of treatment, particularly at concentrations of 1.2, 2.4, and 4.8 μM (Figure 3A), which were selected for further experiments. To assess senescence‐associated β‐galactosidase (SA‐β‐gal) activity, a widely used biomarker of cellular senescence, β‐gal staining was performed. As shown in Figure 3, DOX treatment significantly increased SA‐β‐gal activity in iTECs, while GW9508 administration markedly reduced SA‐β‐gal positivity. At a concentration of 4.8 μM, the proportion of SA‐β‐gal positive cells decreased dramatically from approximately 85% in the DOX‐treated group to about 20%, indicating that GW9508 effectively mitigated cellular senescence (Figure 3B). Additionally, we examined the expression of P21, a canonical senescence marker that inhibits DNA replication and induces irreversible cell‐cycle arrest during aging (Maupin and Adams 2025; You and Wu 2025). Western blot analysis revealed that GW9508 significantly suppressed P21 expression in DOX‐induced senescent iTECs, confirming its anti‐senescent effects (Figure 3C).

To further explore the underlying mechanisms by which GW9508 ameliorates Thymic Aging, gene expression profiles related iTECs function and proliferation were analyzed. The results indicated that GW9508 administration led to a dose‐dependent upregulation of genes associated with iTEC proliferation, such as *CyclinD1*, *FgfR2IIIb*, *CD40*, and FoxN1‐mediated target genes linked to thymocyte development, including *Ccl25*, *Dll4*, *Kit L*, as well as the anti‐apoptotic gene *Survivin* (Figure 3D). Given that Ki67 serves as a nuclear marker for cell proliferation and Aire plays a crucial role in TEC development and the establishment of central immune tolerance (D'Andrea et al. 2025; van Laar et al. 2022), we assessed their expression using flow cytometry, the results showed that the expression levels of both Ki67 and Aire were elevated following GW9508 treatment, indicating that GW9508 enhances cell proliferation and augments the immunoregulatory capacity of senescent iTECs (Figure 3E,F). After treated by GW9508, the subtypes of TECs containing cTECs and mTECs and the expression of CD80 and MHC II molecules were measured by flow cytometry. Flow cytometry gating strategy is shown in Figure S5. The experiment results indicated that GW9508 treatment increased the proportion of cTECs, at the same time, CD80 and MHC II molecules were both upregulated in iTECs (Figure 3G–I). In addition, Annexin V/7‐AAD staining by flow cytometry was performed to evaluate the pro‐apoptotic effect of GW9508 on senescent iTECs. Flow cytometry gating strategy is shown in Figure S6. The results showed that GW9508 could reverse the anti‐apoptotic state of senescent cells, increase the total apoptotic rate of senescent iTECs (Figure 3J), thereby facilitate the clearance of senescent cells.

### 3.4 GW9508 Activated GPR40 to Regulate Its Signaling Pathways, and GW1100 Reversed the Effect of GW9508

**FIGURE 4:**
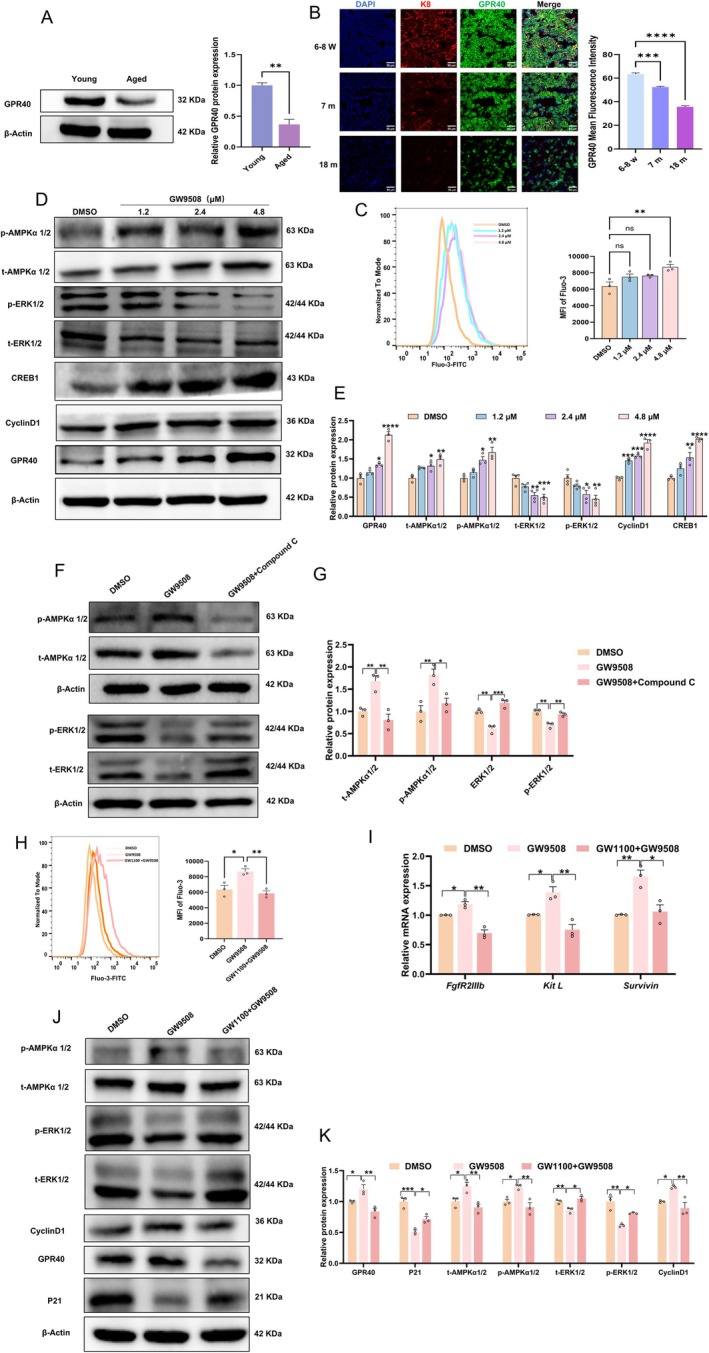
GW9508 activated GPR40 to regulate its signaling pathways, and GW1100 reversed the effect of GW9508. (A) Representative Western blot images and densitometric quantitative analysis of GPR40 expression. (B) Thymic tissues from female mice of different ages (6–8 weeks, 7 months, and 18 months) were prepared as frozen sections, stained with the corresponding antibodies, and imaged by confocal microscopy. Fluorescence intensity was quantified to evaluate GPR40 expression in mice of different ages. Scale bar =50 μm. (C) Representative flow cytometry plots and quantitative analysis of intracellular Ca^2+^ levels in senescent cells following GW9508 treatment for 72 h. (D, E) Representative Western blot images and densitometric quantification of proteins showing the expression of GPR40, P21, CyclinD1 and key proteins in the AMPK and ERK1/2‐MAPK signaling pathways (t‐AMPKα1/2, p‐AMPKα1/2, t‐ERK1/2, and p‐ERK1/2) in senescent cells after GW9508 treatment for 72 h. (F, G) Representative Western blot images and densitometric quantification showing the expression of key proteins in the AMPK and ERK1/2‐MAPK signaling pathways (t‐AMPKα1/2, p‐AMPKα1/2, t‐ERK1/2, and p‐ERK1/2) in senescent cells after GW9508 and the AMPK inhibitor compound C treatment for 72 h (cells were treated with compound C for 1 h, followed by the addition of GW9508 for further incubation of 72 h). (H) Representative flow cytometry plots and quantitative analysis of intracellular Ca^2+^ levels in senescent cells after GW9508 and GW1100 treatment. (GW1100 was treated for 24 h, followed by the addition of GW9508 for further incubation of 48 h). (I) RT‐qPCR analysis of functional gene expression in senescent cells following GW9508 and GW1100 treatment. (GW1100 was treated for 24 h, followed by the addition of GW9508 for further incubation of 48 h). (J, K) Representative Western blot images and densitometric quantification of proteins showing the expression of GPR40, P21, CyclinD1 and key proteins in the AMPK and ERK1/2‐MAPK signaling pathways (t‐AMPKα1/2, p‐AMPKα1/2, t‐ERK1/2, and p‐ERK1/2) in senescent cells after GW9508 and GW1100 treatment. (GW1100 was treated for 24 h, followed by the addition of GW9508 for further incubation of 48 h) All data are presented as mean ± SEM, *n* = 3. Statistical analysis was performed using one‐way ANOVA with Dunnett's multiple comparison test except for the part A in which Two‐tailed independent samples *t*‐test was applied. ns, *p* ≥ 0.05; *p < 0.05, **p < 0.01, ***p < 0.001, ****p < 0.0001.

To evaluate the expression pattern of GPR40 in senescent iTECs, we first compared its protein expression levels between normal and DOX‐induced senescent cells. The results revealed a significant reduction in GPR40 expression in senescent iTECs (Figure 4A). Consistently, immunofluorescence staining of frozen thymic sections from mice of different ages showed a similar trend, with GPR40 expression progressively decreased with advancing age (Figure 4B). Next, senescent iTECs were treated with varying concentrations of GW9508 for 72 h, followed by Western blot analysis. The results demonstrated that GW9508 significantly upregulated the GPR40 expression in senescent cells (Figure 4D). Previous studies have reported that GW9508 activates GPR40, leading to increased intracellular Ca^2+^ levels and subsequent activation of the AMPK signaling pathway (Moonwiriyakit et al. 2018). Our findings aligned this, as GW9508 treatment caused a marked increase in intracellular Ca^2+^ concentration in senescent iTECs (Figure 4C) as well as activation of AMPK and its phosphorylated form (Figure 4D). These observations are consistent with several studies reporting that GPR40 functions through an AMPK pathway‐dependent manner upon activation (Wei et al. 2025; Yun et al. 2025). The ERK1/2‐MAPK pathway is typically associated with cell proliferation, with ERK1/2 signaling promotes cell proliferation in dividing cells but inducing growth arrest in senescent cells (Anerillas et al. 2020). In this study, we further validated this phenomenon, finding that GW9508 treatment reduced the levels of both total ERK1/2 (t‐ERK1/2) and phosphorylated ERK1/2 (p‐ERK1/2). Concurrently, GW9508 treatment increased the expression of downstream proliferation‐related proteins, CyclinD1 and CREB1 (Figure 4D,E). To further clarify the relationship between the AMPK and ERK1/2‐MAPK pathways, we used the AMPK inhibitor compound C to block the AMPK pathway. The results showed that compound C markedly attenuated the activation of the AMPK pathway induced by GW9508, and failed to reverse the excessive activation of the ERK1/2‐MAPK pathway (Figure 4F,G). These findings indicate that the AMPK pathway lies upstream of the ERK1/2‐MAPK pathway, and GW9508 exerts its effects by targeting and activating GPR40, which triggers an increase in intracellular Ca^2+^ concentration, thereby activating the AMPK pathway and subsequently inhibiting the ERK1/2‐MAPK pathway.

To confirm that the effects of GW9508 are mediated through GPR40, additional experimental groups were established. In one set of experiments, the GPR40 inhibitor GW1100 was co‐administered with GW9508. Compared to GW9508 treatment alone, the presence of GW1100 significantly attenuated the ability of GW9508 to activate GPR40, resulting in a reduced rise in intracellular Ca^2+^ levels (Figure 4H). Consequently, the activation of the AMPK pathway was suppressed, and the inhibition of the ERK1/2‐MAPK pathway was weakened. Moreover, the expression of functional genes and proteins was diminished, while P21 expression was notably elevated (Figure 4I–K). These findings indicate that GW1100 effectively antagonizes the actions of GW9508 by specifically inhibiting GPR40.

### 3.5 GPR40 Knockdown Promoted Cellular Senescence, While GPR40 Overexpression Alleviated Cellular Senescence

**FIGURE 5:**
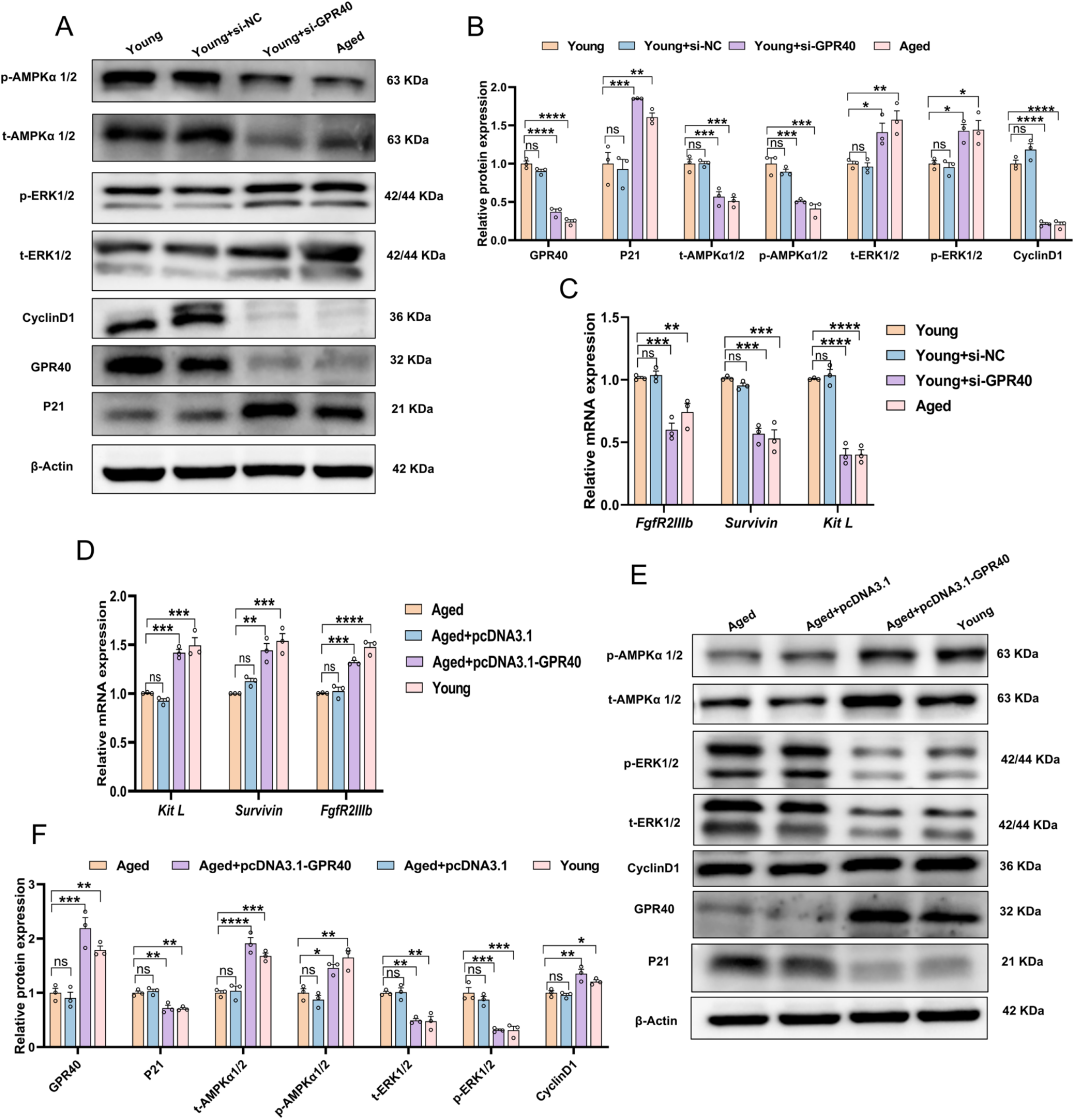
GPR40 knockdown promoted cellular senescence, while GPR40 overexpression alleviated cellular senescence. After validation, the treatment protocol for this section was as follows: SiRNA was transfected into non‐induced young iTECs for 48 h, while pcDNA3.1‐GPR40 was transfected into senescent iTECs for 24 h. (A, B) Representative Western blot images and densitometric quantification of proteins showing the expression of GPR40, P21, CyclinD1 and key proteins in the AMPK and ERK1/2‐MAPK signaling pathways (t‐AMPKα1/2, p‐AMPKα1/2, t‐ERK1/2, and p‐ERK1/2) in GPR40‐knockdown young cells. (C) RT–qPCR analysis of the expression of function‐related genes in GPR40‐knockdown young cells. (D) RT–qPCR analysis of the expression of function‐related genes in senescent cells overexpressing GPR40. (E, F) Representative Western blot images and densitometric quantification of proteins showing the expression of GPR40, P21, CyclinD1 and key proteins in the AMPK and ERK1/2‐MAPK signaling pathways (t‐AMPKα1/2, p‐AMPKα1/2, t‐ERK1/2, and p‐ERK1/2) in senescent cells overexpressing GPR40. All data are presented as mean ± SEM, *n* = 3. Statistical analysis was performed using one‐way ANOVA with Dunnett's multiple comparison test; ns, *p* ≥ 0.05; *p < 0.05, **p < 0.01, ***p < 0.001, ****p < 0.0001.

Building on our preliminary findings, which suggest that the downregulation of GPR40 is associated with cellular senescence, we hypothesized that silencing GPR40 in young cells using si‐*GPR40* could mimic DOX‐induced senescence, driving young cells toward a senescent phenotype. To test this hypothesis, young iTECs were transfected with si‐*GPR40* to knock down GPR40 expression. As expected, GPR40 silencing inhibited the AMPK pathway, exacerbated the aberrant activation of the ERK1/2‐MAPK pathway, downregulated genes and proteins related to cellular function, and increased P21 expression (Figure 5A–C). These results confirm that GPR40 knockdown drives young iTECs toward a senescent state. In contrast, we examined whether the enforced GPR40 expression in senescent cells could replicate the effects of GW9508. To investigate this, senescence was induced in iTECs via DOX treatment, followed by transfection with a *GPR40*‐expression plasmid to elevate GPR40 levels and mimic the action of GW9508. The results showed that GPR40 overexpression in senescent iTECs activated the AMPK pathway while inhibiting the ERK1/2‐MAPK pathway. This was accompanied by an increased expression of functional genes and proteins and a reduction in P21 levels (Figure 5D–F). These findings suggest that restoring GPR40 expression effectively ameliorates the senescent state of iTECs.

## 4 Disscussion

Aging is associated with a gradual decline in tissue and organ function, often resulting in chronic complications and diminished survivability (Khan et al. 2026). The thymus, a pivotal organ of the immune system, functions as the primary site for the development, differentiation, and maturation of T lymphocytes. It consists of thymocytes and thymic stromal cells, with the latter's functional integrity being crucial to thymic function. Among these stromal cells, TECs constitute the predominant population (Gulla et al. 2023; Kadouri et al. 2020; Nitta and Suzuki 2016). During fetal development and early postnatal life, the thymus experiences rapid expansion, reaching its maximum size around puberty. Post‐puberty, however, the thymus gradually undergoes involution, characterized by atrophy, reduced size, diminished functionality, and decreased T‐cell output. These age‐related transformations contribute to immune senescence. During age‐associated thymus involution, thymus size and cellularity undergo a progressive decline accompanied by reduced output of naïve T cells (Srinivasan et al. 2023). Thymic atrophy results in the loss of thymocytes and disruption of thymic architecture, thereby impairing T‐cell development and weakening immune responses to pathogens (Luo et al. 2021). T lymphocytes, the cellular arm of the adaptive immune response, protect the host against foreign pathogens and maintain tissue homeostasis. With aging, T cell functionality declines, leading to compromised immunity against infections, diminished response to vaccination and elevated susceptibility to autoinflammatory diseases and malignancies (Ezuz et al. 2025). This degeneration underlies the decline in immune competence observed in the elderly, who consequently face increased susceptibility to immune‐related diseases, malignancies, and opportunistic infections (Li and Zúñiga‐Pflücker 2023; Liang et al. 2022). As a result, strategies aimed at delaying thymic aging and restoring thymic function have become a major area research, with the ultimate goal of enhancing immune function in aging populations.

In this study, we utilized 17‐month‐old naturally aged female C57BL/6J mice and administered varying doses of GW9508, alongside GW1100, an antagonist with effects opposite to those of GW9508. We evaluated changes in the morphology of the thymus and spleen, organ indices, tissue architecture, hepatic and renal function, thymocyte subsets, TEC subsets, peripheral blood T‐cell subsets, and splenic T‐cell subsets. Compared to the DMSO control group, treatment with GW9508 significantly increased the thymus index and expanded cellular subsets in the thymus, TEC compartment, peripheral blood, and spleen. Conversely, co‐administration of GW1100 with a high dose of GW9508 counteracted most of the beneficial effects observed across measured parameters. These in vivo findings suggest that GW9508 effectively delays thymic aging, promotes thymic restoration, and enhances immune competence in aged mice. Given that GW9508 is a well‐characterized GPR40 agonist and GW1100 serves as its antagonist, the observed antagonism further confirms that GW9508 exerts its biological effects primarily through GPR40, providing a mechanistic basis for subsequent molecular investigations.

Building on the in vivo findings, we further explored the molecular mechanisms underlying GW9508's effects through a series of in vitro assays. Cellular senescence is characterized by growth arrest, reached after a limited number of cell divisions due to the progressive loss of telomere length (replicative senescence), while it could also be a response to various extrinsic genotoxic stimuli, including oxidative stress, oncogenic activation, exposure to chemotherapeutic drugs, and ionizing or ultraviolet (UV) radiation (stress‐induced premature senescence, SIPS) (Fotopoulou et al. 2025; Gorgoulis et al. 2019). One of the commonly used induction methods, D‐galactose‐induced aging primarily operates through mechanisms such as oxidative stress and advanced glycation end product (AGE) formation, which differs from the complex multi‐factorial and multi‐pathway alterations inherent in natural aging (Zhang et al. 2026). DOX is a potent chemotherapy drug commonly used to treat various cancers, including breast cancer, as an anthracycline compound, DOX intercalates into DNA strands to inhibit DNA and RNA synthesis, and this process concurrently triggers DNA double‐strand breaks (DSBs), and inhibition of topoisomerase II, while simultaneously generating reactive oxygen species (ROS), thereby further exacerbating DNA damage and oxidative stress (Bielak‐Zmijewska et al. 2014; Kirsch et al. 2022; Marques et al. 2025; Oda et al. 2023). In addition, DOX‐induced senescence is accompanied by a pronounced senescence‐associated secretory phenotype (SASP), which is closely associated with inflammatory microenvironments (Marques et al. 2025). Given that thymic epithelial cell (TEC) senescence and thymic involution are strongly associated with DNA damage accumulation and chronic inflammation during aging, the DOX‐induced senescence model may better recapitulate the pathological features relevant to our study. Therefore, DOX was selected as a suitable and efficient approach to establish a TEC senescence model in this study. DOX‐induced senescent iTECs were employed as the experimental cell model. A CCK‐8 assay was initially conducted to determine the optimal treatment duration and concentration of GW9508 for enhancing cell viability. These optimized conditions were then applied in subsequent experiments to evaluate various functional and molecular indicators. Our results demonstrate that GW9508 significantly alleviates cellular senescence, as evidenced by a reduced proportion of SA‐β‐gal–positive cells and decreased P21 expression. Moreover, GW9508 upregulated the expression of key functional genes and proteins in senescent iTECs, including *CyclinD1, FgfR2IIIb, CD40, Ccl25, Dll4, Kit L, Survivin*, Aire, and Ki67. Notably, Kit L produced by iTECs can interact with c‐Kit on early T‐cell progenitors (ETPs) to facilitate the transition from DN1 to DN2 stages, corroborating prior findings (Kobayashi et al. 2017). TECs are classified into cortical TECs (cTECs) and medullary TECs (mTECs) according to their anatomical localization (Kondo et al. 2019; Zhang et al. 2025). cTECs are vital for the positive selection of T cells, whereas mTECs are responsible for negative selection, ensuring that mature T lymphocytes recognize self‐major histocompatibility complex (MHC) molecules while maintaining central tolerance (Lancaster et al. 2022; Mishto et al. 2024; Wang et al. 2020). cTECs are essential for selecting thymocytes expressing functional TCRs, mTECs play a key role in central tolerance by the elimination of self‐reactive T‐cells and the differentiation of nonconventional T‐cells, such as regulatory T‐cells (Tregs) (Morales‐Sanchez et al. 2025; Morales‐Sanchez et al. 2023). Previous studies have established that TEC‐derived molecules such as *FOXN1, Ccl25, and Dll4* are critical to early T‐cell maturation and thymic development. *FOXN1*, a pivotal regulatory factor in TEC differentiation and proliferation, is expressed in both cortical and medullary TECs. Age‐associated thymic involution is strongly associated with reduced *FOXN1* expression, which is accompanied by a significant reduction in the expression of its primary downstream targets, *Dll4* and *Ccl25* (Itoi et al. 2006; Li et al. 2023; Liu et al. 2025). During early postnatal thymic development, conditional inactivation of *Dll4* impairs T‐cell differentiation, while inhibition of *Ccl25* disrupts thymocytes migration and activation (Billiard et al. 2011; Williams et al. 2008). Furthermore, *Ccl25* and *Dll4* play crucial roles in the recruitment of early thymic progenitors (ETPs) via immature TECs and facilitate the differentiation of ETPs into the T‐cell lineage (Hozumi et al. 2008; Song et al. 2016; Zlotoff et al. 2010). *CyclinD1* expression in TECs is regulated as a downstream target of the transcription factor FOXN1. The receptor *FgfR2IIIb*, which primarily binds to Fgf7 and Fgf10, is essential for the TECs proliferation (Erickson et al. 2002; Revest et al. 2001). CD80, a costimulatory molecule critical for the activation of CD4^+^ T cells and NK cells (Li, Yang, et al. 2024), along with MHC II, a pivotal antigen‐presenting molecule (Ishina et al. 2023), *CD40* is pivotal not only for maintaining the negative selection function of mTECs but also for promoting their differentiation and proliferation (Akiyama et al. 2015; Hayama et al. 2024). Notably, the defining hallmark of senescent cells is the “persistent viable yet non‐proliferative (zombie)” phenotype. In our experimental setting, the vast majority of severely senescent iTECs were trapped in this irreversible senescent arrest, which requires targeted clearance by pharmacological intervention. Annexin V/7‐AAD flow cytometry revealed that GW9508 functions as a senolytic agent to trigger apoptosis and eliminate severely senescent iTECs. Meanwhile, a small fraction of mildly senescent iTECs achieved functional recovery following GW9508 administration. Mechanistically, upregulated *Survivin* together with increased Aire and Ki67 expression facilitated the recovery of senescent iTECs, strengthening their proliferation potential and physiological functions in mediating thymic negative selection and central immune tolerance construction.

GW9508 has been identified as a potent and effective agonist of GPR40, primarily activating intracellular signaling mediated by the Gq family of G proteins, leading to increased intracellular calcium levels (Defossa and Wagner 2014; Watkins and Orlandi 2020). Numerous studies have confirmed that GPR40 functions as a Gq/11‐coupled receptor, initiating phospholipase C (PLC)‐dependent signaling and subsequently elevating intracellular Ca^2+^ levels (Briscoe et al. 2003; Fujiwara et al. 2005; Itoh et al. 2003; Suh et al. 2008). Consistent with these findings, our Western blot and confocal microscopy analyses revealed a significant reduction in GPR40 expression in DOX induced senescent iTECs, alongside an age‐related decline in GPR40 levels within thymic tissues. Notably, treatment of senescent cells with GW9508 restored GPR40 expression, and as previously reported, administration of GW9508 increased intracellular Ca^2+^ levels. These findings suggest that GW9508 exerts its effects through direct targeting and activation of GPR40.

Furthermore, several studies have demonstrated that GPR40 activation stimulates the AMPK signaling pathway (Lei et al. 2024; Yun et al. 2025). For instance, prior research reported that GW9508 treatment enhances tight junction assembly in Calu‐3 airway epithelial cells and improves epithelial barrier function, an effect attributed to the calcium‐dependent activation of the AMPK pathway following GPR40 activation (Moonwiriyakit et al. 2018). Similarly, Fan et al. (2024) demonstrated that administering exogenous nucleotides (NTs) to mice with accelerated aging activated the AMPK pathway and suppressed the MAPK pathway, thereby modulating autophagy and mitigating skin aging. Likewise, Sun et al. (2011) found that ω‐3 fatty acids inhibit ERK1/2 phosphorylation in breast cancer cells, likely mediated through GPR40 activation, as ω‐3 fatty acids serve as endogenous agonists of this receptor. Previous studies also demonstrated that under inflammatory conditions, GW9508 prevents cytokine‐induced airway epithelial barrier disruption by inhibiting ERK1/2 phosphorylation via a PLC‐ and calcium/calmodulin‐dependent protein kinase β (CaMKKβ)‐dependent mechanism (Sone and Takayanagi 1983).

Consistent with these findings, our experimental model of cellular senescence, frequently associated with a pro‐inflammatory state, revealed a phenomenon known as “inflammaging”. This condition significantly contributes to organismal aging, as the age‐related decline in the immune system's ability to clear senescent cells and pro‐inflammatory mediators progressively elevates inflammatory levels, exacerbating aging‐related damage (Singh et al. 2024). Based on these observations, we propose a mechanistic hypothesis wherein GW9508 mitigates thymic aging by targeting and activating GPR40, subsequently stimulating the AMPK pathway while inhibiting the ERK1/2‐MAPK pathway.

To test this hypothesis, we examined the expression of key signaling proteins implicated in this mechanism. Consistent with our hypothesis, GW9508 treatment activated of the AMPK pathway and suppressed the ERK1/2‐MAPK pathway in senescent cells, accompanied by increased expression of downstream proliferative proteins CyclinD1 and CREB1. To further confirm that GW9508 exerts its effects via GPR40, we established additional experimental groups, including a GPR40 inhibitor (GW1100), GPR40 knockdown, and GPR40 overexpression. The results demonstrated that inhibition of GPR40 by GW1100 counteracted the effects of GW9508, consistent with our in vivo findings. GPR40 knockdown suppressed the AMPK pathway and exacerbated ERK1/2‐MAPK pathway activation, promoting a senescent phenotype in young cells, whereas GPR40 overexpression produced the opposite effect, alleviating cellular senescence. Collectively, these results demonstrate that GW9508 targets GPR40 to activate the AMPK pathway while inhibiting the ERK1/2‐MAPK pathway, thereby exerting anti‐senescent effects.

Nonetheless, the absence of a young mouse control group and histopathological evaluation of the spleen are limitations of this study, and these will be addressed in future investigations, which can more intuitively verify the reversing effect of GW9508 on age‐related thymic dysfunction. To date, research on GW9508 has primarily focused on its role in insulin metabolism and diabetes‐related disorders. Our research highlights a previously unrecognized function of GW9508 in alleviating thymic aging and improving immune function in aged individuals. These findings offer novel insights into the translational potential of GW9508 and lay the groundwork for its future development as an anti‐aging therapeutic agent.

## Author Contributions

Yinhong Song: conceptualization, methodology, validation, formal analysis, investigation, data curation, writing – original draft, writing – reviewing and editing, visualization, project administration; Qingqing Li, Pin Zhu: methodology, validation, formal analysis, investigation, writing – review and editing; Lihong Cui, Fenghua Luo: investigation, data curation, writing – review and editing; Yongqin Zhou, Yuanyuan Hu, Chunyan Luo, Shanshan Han: investigation, data curation; Xie Ding, Maoxi Yao: validation, formal analysis; Honggang Yuan: conceptualization, investigation, writing – review and editing, supervision, project administration, funding acquisition. All authors read and approved the final manuscript.

## Funding

This study was supported by the National Natural Science Foundation of China (No. 81671397), Hubei Provincial Natural Science Foundation of China (No. 2024AFD127), the scientific project of Health Commission of Hubei Province of China (No. WJ2025M025), the Open Foundation of Hubei Provincial Key Laboratory of Tumor Microenvironment and Immunotherapy in China (No. 2024ZLKF2‐56), and Science Project of Yichang Municipal Science and Technology Bureau of Hubei Province in China (A25‐4‐014 and A22‐2‐016).

## Consent

All authors concur with the submission and publication of this paper.

## Conflicts of Interest

The authors declare no conflicts of interest.

## Supporting information


**Figure S1:** Analytical strategy and representative figures for thymocyte subsets detection by flow cytometry.
**Figure S2:** Analytical strategy and representative figures for the detection of thymic epithelial cells (TECs) and their subsets by flow cytometry.
**Figure S3:** Analytical strategy and representative figures for the detection of splenic T lymphocytes and their subsets by flow cytometry.
**Figure S4:** Analytical strategy and representative figures for the detection of peripheral blood T lymphocytes and their subsets by flow cytometry.
**Figure S5:** Analytical strategy for detecting changes in cell subsets after GW9508 treatment for 72 h by flow cytometry.
**Figure S6:** Analytical strategy for detecting cell apoptosis after GW9508 treatment for 72 h by flow cytometry.
**Figure S7:** Immunofluorescence staining was performed to detect GPR40 expression in mouse thymic tissues following drug treatment.
**Figure S8:** Cells were identified via immunofluorescence staining for K8 and K5.
**Table S1:** Primer sequences for the amplification.

## Data Availability

The data that support the findings of this study are available from the corresponding author upon reasonable request.
