# Supplementary material for: GW9508‐Induced Activation of GPR40 in Thymic Epithelial Cells: A Therapeutic Strategy to Delay Thymic Aging
Source: Aging Cell. 2026 Jul 13;25(7):e70630. doi: 10.1111/acel.70630 (PMC13365366; doi:10.1111/acel.70630)
Supplement: Supplementary file 1 — Figure S1: Analytical strategy and representative figures for thymocyte subsets detection by flow cytometry. Figure S2: Analytical strategy and representative figures for the detection of thymic epithelial cells (TECs) and their subsets by flow cytometry. Figure S3: Analytical strategy and representative figures for the detection of splenic T lymphocytes and their subsets by flow cytometry. Figure S4: Analytical strategy and representative figures for the detection of peripheral blood T lymphocytes and their subsets by flow cytometry. Figure S5: Analytical strategy for detecting changes in cell subsets after GW9508 treatment for 72 h by flow cytometry. Figure S6: Analytical strategy for detecting cell apoptosis after GW9508 treatment for 72 h by flow cytometry. Figure S7: Immunofluorescence staining was performed to detect GPR40 expression in mouse thymic tissues following drug treatment. Figure S8: Cells were identified via immunofluorescence staining for K8 and K5. Table S1: Primer sequences for the amplification. [file ACEL-25-e70630-s001.docx]

**Supplementary Materials for**

**GW9508-Induced Activation of GPR40 in Thymic Epithelial Cells: A Therapeutic Strategy to Delay Thymic Aging**

Qingqing Li^1,2,3†^, Ping Zhu^4†^, Lihong Cui^1,2,3^, Fenghua Luo^1,2,3^, Yongqin Zhou^1,2,3^, Yuanyuan Hu^1,2,3^, Chunyan Luo^1,2,3^, Shanshan Han^1,2,3^, Xie Ding^1,2,3^, Maoxi Yao^1,2,3^, Honggang Yuan^5*^, Yinhong Song^1,2,3*^

^1. Hubei Key Laboratory of Tumor Microenvironment and Immunotherapy, China Three Gorges University, Yichang 443002, China^

^2. Institute of Infection and Inflammation, China Three Gorges University, Yichang 443002, China^

^3. College of Basic Medical Sciences, China Three Gorges University, Yichang 443002, China^

^4. Department of Nephrology, The First College of Clinical Medical Science, China Three Gorges University, Yichang Center People's Hospital, Yichang, 443002, China^

^5. Department of Urology, The First College of Clinical Medical Science, China Three Gorges University, Yichang Center People's Hospital, Yichang, 443002, China^

^†^Qingqing Li, Ping Zhu contributed equally to this work

* Correspondence:

Yinhong Song ORCID: 0000-0003-4136-8744. The full address: China Three Gorges University, 8 Daxue Road, Xiling District, Yichang city, Hubei Province, 443002, China. E-mail:syh728@ctgu.edu.cn. Phone：+8613986832645. Fax: (+86)0717-6397348.

Honggang Yuan ORCID: 0000-0003-1550-3359. The full address: China Three Gorges University, 8 Daxue Road, Xiling District, Yichang city, Hubei Province, 443002, China. E-mail：2820252006@qq.com. Phone：+8615171742999. Fax: (+86)0717-6488564.

**This file includes:**

Supplementary Figure 1 : Analytical strategy and representative figures for thymocyte subsets detection by flow cytometry.

Supplementary Figure 2 : Analytical strategy and representative figures for the detection of thymic epithelial cells (TECs) and their subsets by flow cytometry.

Supplementary Figure 3 : Analytical strategy and representative figures for the detection of splenic T lymphocytes and their subsets by flow cytometry.

Supplementary Figure 4 : Analytical strategy and representative figures for the detection of peripheral blood T lymphocytes and their subsets by flow cytometry.

Supplementary Figure 5 : Analytical strategy for detecting changes in cell subsets after GW9508 treatment for 72 h by flow cytometry.

Supplementary Figure 6 : Analytical Strategy for Detecting Cell Apoptosis after GW9508 Treatment for 72 h by Flow Cytometry.

Supplementary Figure 7 : Immunofluorescence staining was performed to detect GPR40 expression in mouse thymic tissues following drug treatment.

Supplementary Figure 8 : Cells were identified via immunofluorescence staining for K8 and K5.

Supplementary Table 1 : Primer Sequences for the Amplification


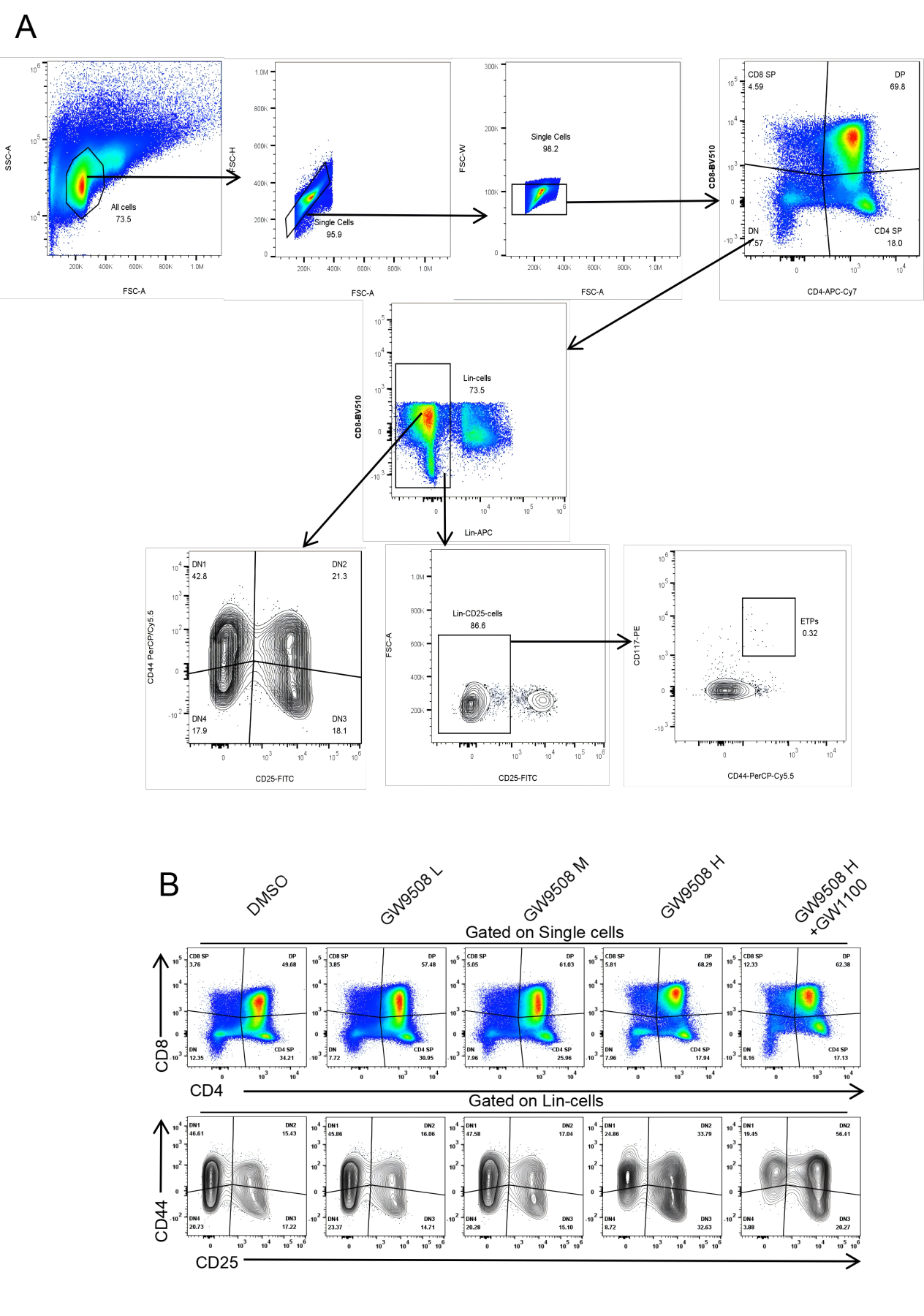


**Supplementary Figure 1 : Analytical strategy and representative figures for thymocyte subsets detection by flow cytometry.** (A) Gating strategy for flow cytometric identification of thymocytes and their subsets in animal experiments ; (B) Representative flow cytometry plots showing thymocyte subsets in aged mice, including DN (CD4- CD8-), DP (CD4^+^ CD8^+^), CD8^+^ SP (CD4^-^ CD8^+^), CD4^+^ SP (CD4^+^ CD8^-^), DN1 (Lin^−^ CD25^−^ CD44^+^), DN2 (Lin^−^ CD25^+^ CD44^+^), DN3 (Lin^−^ CD25^+^ CD44^−^) and DN4 (Lin^−^ CD25^−^ CD44^−^) subsets.


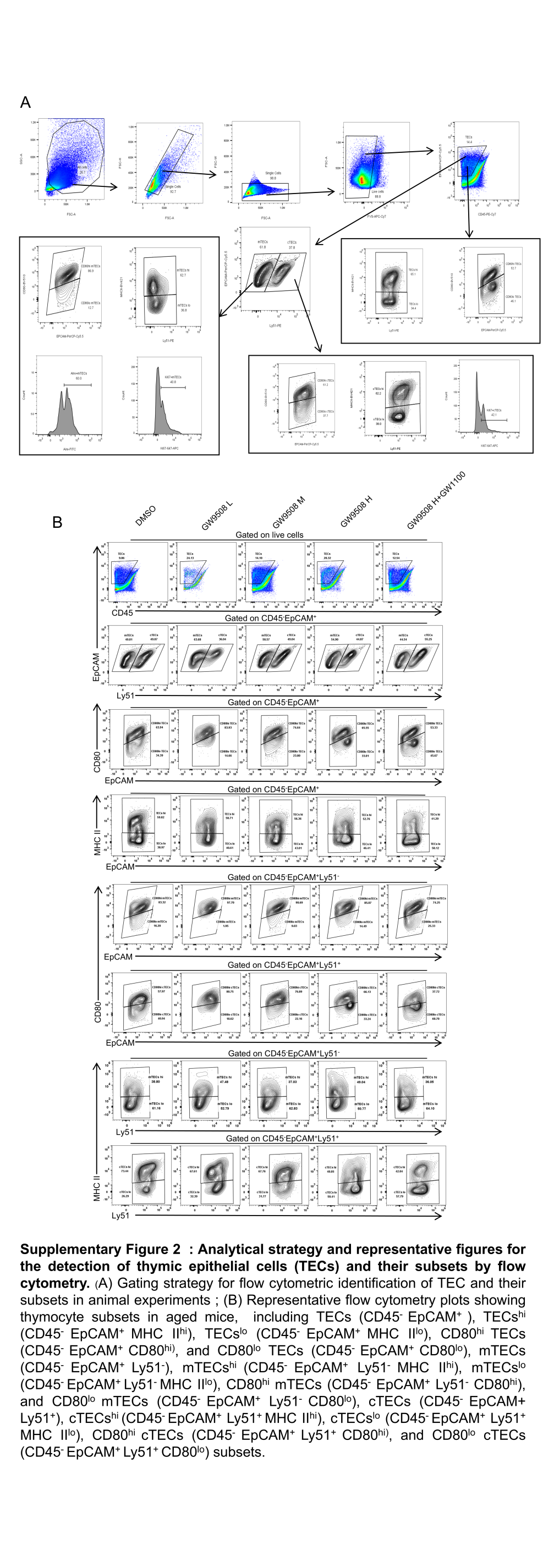


**Supplementary Figure 2 : Analytical strategy and representative figures for the detection of thymic epithelial cells (TECs) and their subsets by flow cytometry.** (A) Gating strategy for flow cytometric identification of TECs and their subsets in animal experiments ; (B) Representative flow cytometry plots showing thymocyte subsets in aged mice, including TECs (CD45^-^ EpCAM^+^ ), TECs^hi^ (CD45^-^ EpCAM^+^ MHC II^hi^), TECs^lo^ (CD45^-^ EpCAM^+^ MHC II^lo^), CD80^hi^ TECs (CD45^-^ EpCAM^+^ CD80^hi^), and CD80^lo^ TECs (CD45^-^ EpCAM^+^ CD80^lo^), mTECs (CD45^-^ EpCAM^+^ Ly51^-^), mTECs^hi^ (CD45^-^ EpCAM^+^ Ly51^-^ MHC II^hi^), mTECs^lo^ (CD45^-^ EpCAM^+^ Ly51^-^ MHC II^lo^), CD80^hi^ mTECs (CD45^-^ EpCAM^+^ Ly51^-^ CD80^hi^), and CD80^lo^ mTECs (CD45^-^ EpCAM^+^ Ly51^-^ CD80^lo^), cTECs (CD45^-^ EpCAM^+^ Ly51^+^), cTECs^hi^ (CD45^-^ EpCAM^+^ Ly51^+^ MHC II^hi^), cTECs^lo^ (CD45^-^ EpCAM^+^ Ly51^+^ MHC II^lo^), CD80^hi^ cTECs (CD45^-^ EpCAM^+^ Ly51^+^ CD80^hi^), and CD80^lo^ cTECs (CD45^-^ EpCAM^+^ Ly51^+^ CD80^lo^) subsets.


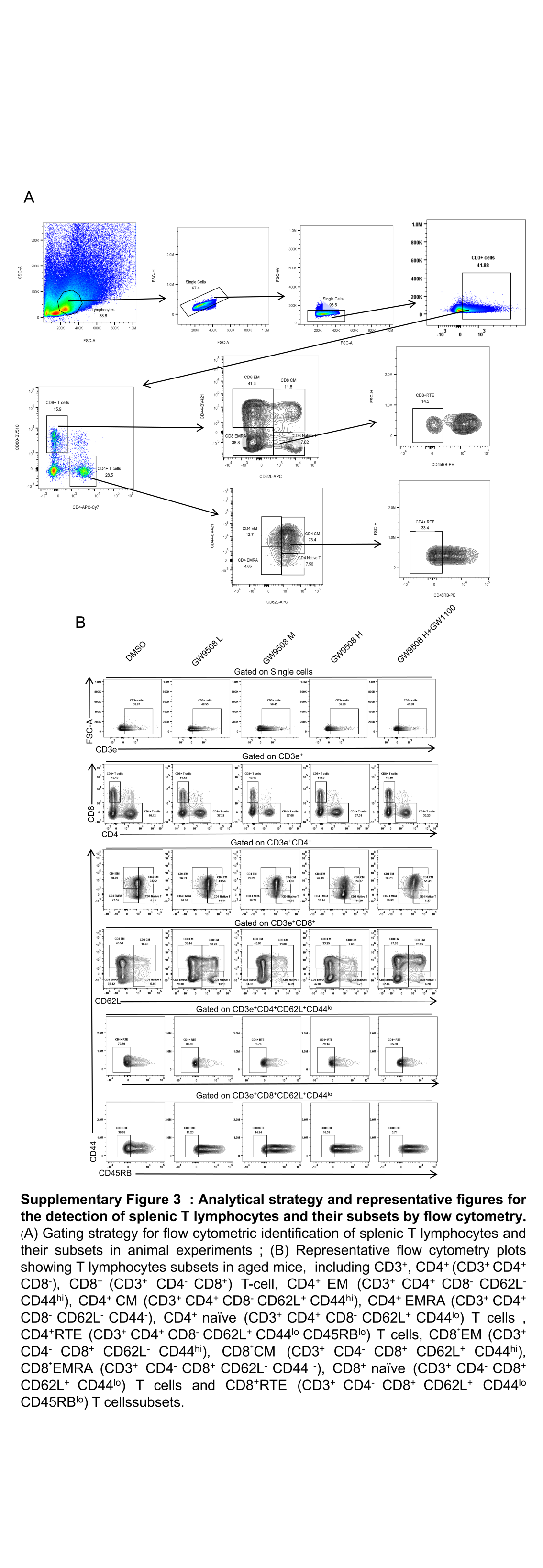


**Supplementary Figure 3 : Analytical strategy and representative figures for the detection of splenic T lymphocytes and their subsets by flow cytometry.** (A) Gating strategy for flow cytometric identification of splenic T lymphocytes and their subsets in animal experiments ; (B) Representative flow cytometry plots showing T lymphocyte subsets in aged mice, including CD3^+^, CD4^+^ (CD3^+^ CD4^+^ CD8^-^), CD8^+^ (CD3^+^ CD4^-^ CD8^+^) T cell, CD4^+^ EM (CD3^+^ CD4^+^ CD8^-^ CD62L^-^ CD44^hi^), CD4^+^ CM (CD3^+^ CD4^+^ CD8^-^ CD62L^+^ CD44^hi^), CD4^+^ EMRA (CD3^+^ CD4^+^ CD8^-^ CD62L^-^ CD44^-^), CD4^+^ naïve (CD3^+^ CD4^+^ CD8^-^ CD62L^+^ CD44^lo^) T cells , CD4⁺RTE (CD3^+^ CD4^+^ CD8^-^ CD62L^+^ CD44^lo^ CD45RB^lo^) T cells, CD8⁺EM (CD3^+^ CD4^-^ CD8^+^ CD62L^-^ CD44^hi^), CD8⁺CM (CD3^+^ CD4^-^ CD8^+^ CD62L^+^ CD44^hi^), CD8⁺EMRA (CD3^+^ CD4^-^ CD8^+^ CD62L^-^ CD44 ^-^), CD8^+^ naïve (CD3^+^ CD4^-^ CD8^+^ CD62L^+^ CD44^lo^) T cells and CD8⁺RTE (CD3^+^ CD4^-^ CD8^+^ CD62L^+^ CD44^lo^ CD45RB^lo^) T cellssubsets.


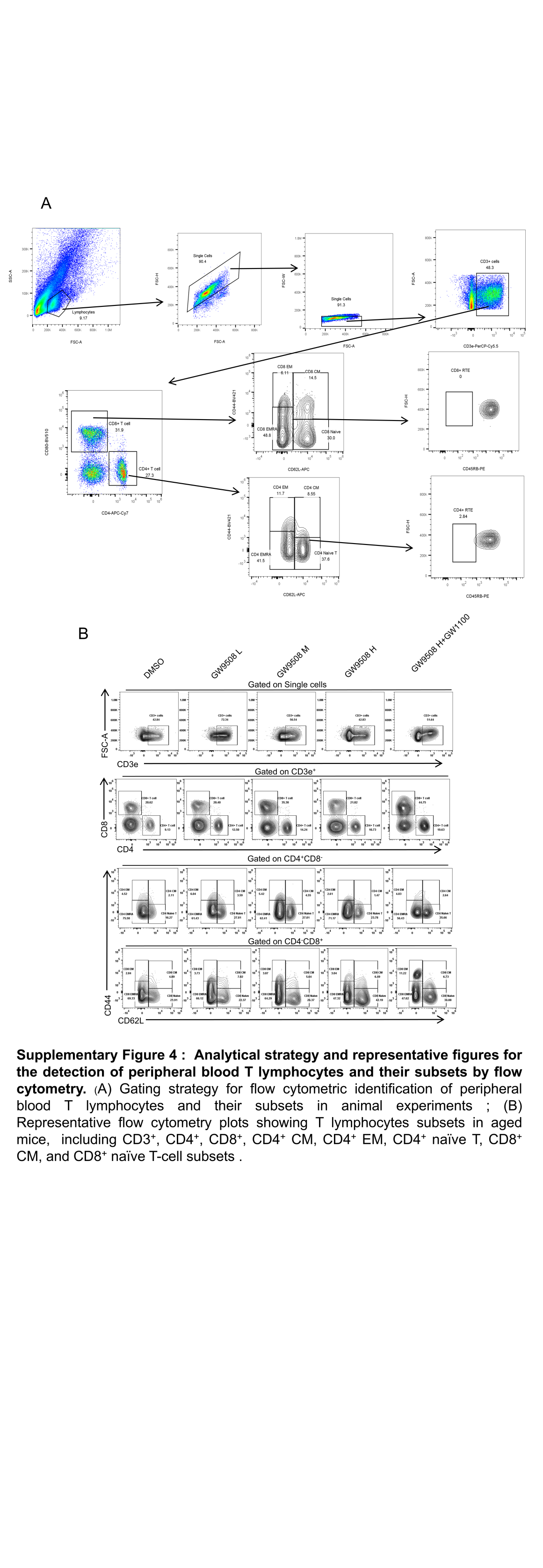


**Supplementary Figure 4 : Analytical strategy and representative figures for the detection of peripheral blood T lymphocytes and their subsets by flow cytometry.** (A) Gating strategy for flow cytometric identification of peripheral blood T lymphocytes and their subsets in animal experiments ; (B) Representative flow cytometry plots showing T lymphocytes subsets in aged mice, including CD3^+^, CD4^+^, CD8^+^, CD4^+^ CM, CD4^+^ EM, CD4^+^ naïve T, CD8^+^ CM, and CD8^+^ naïve T-cell subsets .





**Supplementary Figure 5 : Analytical strategy for detecting changes in cell subsets after GW9508 treatment for 72 h by flow cytometry.** (A) Gating strategy for flow cytometric identification of iTECs and their subsets in cell experiments ; (B) Representative flow cytometry plots showing iTECs subsets in senescent cells , including cTECs (CD45^-^ EpCAM^+^ Ly51^+^), TECs^hi^ (CD45^-^ EpCAM^+^ MHC II^hi^), and CD80^hi^ TECs (CD45^-^ EpCAM^+^ CD80^hi^) subsets.





**Supplementary Figure 6 : Analytical Strategy for Detecting Cell Apoptosis after GW9508 Treatment for 72 h by Flow Cytometry.** Q1: necrotic cells; Q2: late apoptotic cells; Q3: early apoptotic cells; Q4: viable cells.





**Supplementary Figure 7: Immunofluorescence staining was performed to detect GPR40 expression in mouse thymic tissues following drug treatment.** Blue indicates DAPI for nuclear staining, red indicates K8 for labeling cortical thymic epithelial cells, and green indicates GPR40. After merging, yellow fluorescence indicates the expression of GPR40 in the thymus.





**Supplementary Figure 8: Cells were identified via immunofluorescence staining for K8 and K5.** Mouse leukemia L1210 cells lacking K8/K5 expression served as the negative control. Nuclei were counterstained blue with DAPI. Red fluorescence indicated cortical thymic epithelial cells labeled with K8, and green fluorescence represented medullary thymic epithelial cells labeled with K5.

**Supplementary Table 1 : Primer Sequences for the Amplification**
